# Implementation Determinants and Outcomes of a Technology-Enabled Service Targeting Suicide Risk in High Schools: Mixed Methods Study

**DOI:** 10.2196/16338

**Published:** 2020-07-20

**Authors:** Molly Adrian, Jessica Coifman, Michael D Pullmann, Jennifer B Blossom, Casey Chandler, Glen Coppersmith, Paul Thompson, Aaron R Lyon

**Affiliations:** 1 Department of Psychiatry and Behavioral Sciences University of Washington Seattle, WA United States; 2 Seattle Children's Research Institute Seattle, WA United States; 3 Qntfy Arlington, VA United States; 4 The Dartmouth Institute for Health Policy and Clinical Practice Geisel School of Medicine at Dartmouth Hanover, NH United States

**Keywords:** technology-enabled services, suicide prevention, school-based mental health, user-centered design, mobile phone

## Abstract

**Background:**

Technology-enabled services (TESs), which integrate human service and digital components, are popular strategies to increase the reach and impact of mental health interventions, but large-scale implementation of TESs has lagged behind their potential.

**Objective:**

This study applied a mixed qualitative and quantitative approach to gather input from multiple key user groups (students and educators) and to understand the factors that support successful implementation (implementation determinants) and implementation outcomes of a TES for universal screening, ongoing monitoring, and support for suicide risk management in the school setting.

**Methods:**

A total of 111 students in the 9th to 12th grade completed measures regarding implementation outcomes (acceptability, feasibility, and appropriateness) via an open-ended survey. A total of 9 school personnel (school-based mental health clinicians, nurses, and administrators) completed laboratory-based usability testing of a dashboard tracking the suicide risk of students, quantitative measures, and qualitative interviews to understand key implementation outcomes and determinants. School personnel were presented with a series of scenarios and common tasks focused on the basic features and functions of the dashboard. Directed content analysis based on the Consolidated Framework for Implementation Research was used to extract multilevel determinants (ie, the barriers or facilitators at the levels of the outer setting, inner setting, individuals, intervention, and implementation process) related to positive implementation outcomes of the TES.

**Results:**

Overarching themes related to implementation determinants and outcomes suggest that both student and school personnel users view TESs for suicide prevention as moderately feasible and acceptable based on the Acceptability of Intervention Measure and Feasibility of Intervention Measure and as needing improvements in usability based on the System Usability Scale. Qualitative results suggest that students and school personnel view passive data collection based on social media data as a relative advantage to the current system; however, the findings indicate that the TES and the school setting need to address issues of privacy, integration into existing workflows and communication patterns, and options for individualization for student-centered care.

**Conclusions:**

Innovative suicide prevention strategies that rely on passive data collection in the school context are a promising and appealing idea. Usability testing identified key issues for revision to facilitate widespread implementation.

## Introduction

### Background

Suicide is the second leading cause of death for adolescents, and the rate of suicide in the United States has increased in recent years [1,2]. Suicidal thoughts and behaviors (including suicidal ideation, nonsuicidal self-injury, and suicide attempts) increase dramatically during adolescence and are unfortunately common. Data suggest that 17% of US high school students seriously consider suicide each year, and approximately 9% report a suicide attempt [3]. Despite this being a common health concern, our ability to predict suicide is poor [4].

High schools provide a convenient and accessible setting to promote mental health, as most children attend high schools, removing practical barriers for mental health services and promoting care for traditionally underserved groups [5-9]. Thus, schools have the potential to play an essential role in supporting the identification and treatment of youth with mental health difficulties. School is also the most common community setting where suicidal ideation is identified [10]; however, the majority of schools demonstrate poor adherence to gold standard practices related to the identification and treatment of youth at risk [11,12]. Many high schools do not adopt recommended suicide prevention strategies because of practical concerns, such as the capacity to manage false positives, lack of knowledge of evidence-based practices, fear, stigma, as well as legal and ethical issues [11,13].

Technology-enabled services (TESs) hold promise for adolescent suicide prevention because of their capacity to support best practices while achieving population health and may help address many of the practical concerns that act as barriers to the adoption of suicide prevention strategies (ie, cognitive load, burden, and costs). TESs are characterized by having both a human service component (eg, therapist-delivered psychotherapies) and a digital component (eg, dashboard app) that supports, or is supported by, the service [14]. A broad range of support falls under the category of TES, including web- or app-based supports for posttraumatic stress disorder treatment (eg, Prolonged Exposure Coach), interventions for insomnia (Cognitive Behavioral Therapy for Insomnia Coach), and substance use disorders (reSET) [15,16]. Efficacy trials indicate that TESs yield effects commensurate with well-established psychological interventions for depression, anxiety, and suicidal ideation/behavior [17-19]. Nearly all teens are heavy users of smartphones and other digital technologies and use these technologies for health-related concerns [20]. Furthermore, adolescents are seeking TES to manage their health [21]. TES via text-based extensions (eg, Text4Strength with Sources of Strength) of suicide prevention programs show initial feasibility, safety, and utility from the adolescent perspective [22].

High schools represent a key setting for which TES can be effectively adapted and applied. An important suicide prevention research priority is to evaluate how TES may address many of the challenges that schools face in adopting suicide prevention strategies. For example, TES that automatically and continuously monitors social media (SM) data and requires virtually no staff or resources such as time in the classroom to execute may be valuable in reducing the burden of universal screening. Furthermore, strategies that rely on student-generated SM data may reduce common concerns about specific suicide or emotional health screening tools [23,24]. Furthermore, as TES does not rely on explicit reports of suicidal ideation or behavior, the potential for student stigmatization is substantially reduced. A platform that allows for passive data aggregation and monitoring is ongoing rather than linked to one particular assessment time point, facilitating an identification approach that aligns with the episodic nature of suicidality [23-25]. For these reasons, strategic monitoring of SM, has the potential to have a considerable impact on public health via scalable early detection and intervention to decrease adolescent suicide rates.

Although research on the efficacy of TES for mental health broadly—and suicide prevention specifically—is promising [21], school settings have experienced few benefits from TES. Much of this is likely because of insufficient attention to (1) end users’ priorities and experiences regarding aspects of the technology and the human service and (2) the implementation strategies that promote their adoption and sustained use [14,26]. To improve TES implementation, developers are increasingly turning to the methods of *human-centered design (HCD)* to identify and address problematic system design and its impact on otherwise appealing and effective products [27]. HCD includes a set of approaches that ground the development process in information about the needs and desires of people who will ultimately use a product, with the goal of creating compelling, intuitive, and effective innovations [28,29]. Usability testing, a hallmark of HCD, provides an opportunity for representative end users to interact with the technology, complete specific tasks, and generate information about the functionality and presentation. Owing to the potential impact of TES usability on implementation outcomes [30], we prioritized this determinant in the study design.

### Objectives

The goal of this research was to evaluate the implementation outcomes of acceptability, feasibility, and appropriateness from the primary users of a TES (Quinn Therapeutic) for school-based suicide prevention (ie, school staff and students). These perceived implementation outcomes are critical precursors to adoption and use [30] and can be most effectively assessed at early project stages before actual implementation occurs [31]. As articulated by Proctor et al [31], acceptability is defined as perceptions that an innovation is agreeable, palatable, or satisfactory; feasibility is the extent to which a new innovation can be successfully used or carried out within a given agency or setting; and appropriateness refers to the innovation’s fit, relevance, and compatibility with the setting, staff, and target problem. We also evaluated implementation determinants, including usability factors, driving our primary implementation outcomes. Our specific research questions were as follows: (1) What key, multilevel factors in the school context should drive the adaptation and implementation of a student-monitoring dashboard interface of the Quinn Therapeutic and (2) What changes to the digital dashboard interface are needed to maximize its acceptability, feasibility, appropriateness, and ultimate usability for school systems? We focus on the dashboard interface for applying HCD as the most salient and user-facing feature of TES.

## Methods

### Participants and Procedures

Participants were drawn from an urban area in the Pacific Northwest. Recruitment of participants occurred through past and ongoing research partnerships. Interested principals and/or school counselor leads were presented with the information regarding study procedures in an initial meeting and then recontacted if they were interested in having students and school personnel the opportunity to participate. Recruitment of clinician participants was done in the winter of 2018, with user testing procedures completed in February and March; student recruitment and study student data collection procedures were performed in May 2018. All procedures were approved by our institutional review board (study 3246).

#### Procedures for School Personnel: User Testing

In total, 9 school personnel whose responsibilities are most proximal to school-based suicide screening—school nurses, counselors, social workers, and administrators—were invited to participate in the laboratory-based user testing of our student-monitoring dashboard.

School personnel were included to identify key aspects of the Quinn Therapeutic system that were most in need of redesign. Drawing from established models of user testing [32], participants were presented with a series of scenarios that contain common tasks to accomplish. Tasks focused on the primary features of the dashboard system (ie, open exploration, identifying a student at risk for suicide, and identifying the risk status of a new student). During the task completion, participants used a *think-aloud* data collection technique [33], describing their processes and experiences as they navigate the system. Anticipated and actual task difficulties were assessed consistent with Albert and Dixon’s [34] method. During system testing, participants rated on a 5-point scale (ranging from 1=*very easy* to 5=*very difficult*) the expected difficulty of each task. Following task completion, participants rated the experienced difficulty on the same scale. We used prompts such as *What are you thinking about? What details are you looking for?* and *What is your impression of this task* to elicit information. Following the completion of the task and their post-task rating, we asked several follow-up questions including, *Why did you answer the way you did? Was there anything confusing, usual, or difficult to understand about this task*? as well as additional questions specific to individualized tasks. Each session concluded with a qualitative open-ended interview to gather additional feedback about the system and implementation determinants as well as completion of standardized measures of implementation outcomes and system usability (system usability scale; SUS [35]).

#### Procedures for Students: Social Media Data and Survey

Following school administrator approval, 111 students were recruited from a private high school in an urban area of the Pacific Northwest. Students were eligible to participate if they attended high school and used 1 of 5 popular SM platforms (Twitter, Facebook, Instagram, Reddit, and Tumblr) on a weekly basis.

The opportunity for participation was presented in an all-class assembly. A brief orientation to the procedures was given, and assent was obtained from youth. Parents/caregivers received a letter from the principal informing of study procedures and the option to opt out their high school from the study procedures. Youth who provided consent were given access to the University of Washington OurDataHelps website to donate SM data and complete questionnaires regarding their preferences for the Quinn Therapeutic dashboard. Students opted for study participation through the OurDataHelps website [36]. This website provided study information and provided a web-based data collection platform. The components of student data collection included SM data donation, and questionnaires regarding emotional health, implementation outcomes, and SM use and preferences were completed via a web or mobile platform. Following the presentation, eligible students were given 1 week to access the survey platform and complete study procedures. Students received US $30 for participation.

### Materials: Quinn Therapeutic Dashboard

Quinn Therapeutic is a TES that aggregates patient-generated SM data to detect and monitor suicide risk, visualize data over time, and provide feedback to clinicians, which may be particularly suitable in the high school context [37-39]. Quinn Therapeutic’s core digital and human service features are outlined below (Table 1). The technology relies on deep learning (a subset of machine learning algorithms), which enable a computer to discover and use patterns in data, trained, and optimized using SM data [40]. The aggregated SM data and risk ratings based on machine learning algorithms are presented in a student-monitoring dashboard interface, which clinicians log into to view estimated student risk status, data over time, and SM content driving ratings of risk. The dashboard’s purpose is to allow school personnel to monitor student suicide risk over time. Core functions are represented in the web-based supplement. A timeline shows the overall data contribution for the population; there is a search function for finding specific students, and tabs categorize students with high risk for ease of viewing the at-risk population with past suicidal ideation and self-harm. School personnel can also view individuals, including their suicide risk level over time (ie, risk level graph), source SM data that generate the risk ratings, the strength and valence of sentiment (ie, sentiment graph), and students self-reported diagnostic and suicide risk information. Quinn Therapeutic’s predictive algorithms have been developed and evaluated in users who donate their data to an online portal as well as publicly available data from Twitter and demonstrated impressive accuracy in distinguishing those with identifying self-reported suicide attempts from those who did not report this history [41]. The algorithms demonstrated the capability to separate users who would attempt suicide from neurotypical controls. Evaluating SM data from the month before a suicide attempt, the area under the curve (AUC) from receiver operating characteristics for this binary decision task was 0.89, and for all available SM data, AUC was 0.94 (an AUC of 1 is perfect prediction) [42]. Quinn Therapeutic human service components include measurement-based care, crisis prevention planning, and risk management, all of which align with recommendations for the identification and management of adolescent suicide risk [43]. As a first step in evaluating the application of Quinn Therapeutic TES in the school setting, the current project focused only on an early prototype of Quinn Therapeutic’s digital technology component.

**Table 1:** Quinn Therapeutic technology specifications.

Data aggregation		Ongoing data capture from five SM^a^ platforms—Facebook, Instagram, Twitter, Reddit, and Tumblr
**Digital platform components**		
	Risk prediction	Relies on *deep learning*, specifically refined for suicide-specific predictions from multiple cohorts
	Visualization of progress	Student-monitoring dashboard interface for selected school personnel
	Data security and privacy	Health Insurance Portability and Accountability Act–compliant cloud-based server, opt in participation, and meets recommendations for ethical use of SM data [44]
**Service components**		
	Measurement-based care	Ongoing monitoring through passive data collection
	Crisis prevention planning	Use of real-time data to understand past prompting events and plan for future
	Suicide-specific assessment and treatment	Maintains top priority of safety at the time it is needed

^a^SM: social media.

For user testing sessions, school personnel viewed the student-monitoring dashboard populated with *dummy data*. The dashboard allows for data visualization of the posterior probability (range 0-1) representing the likelihood that each individual SM post was written by someone at risk of suicide and status updates that were analyzed with machine learning algorithms developed in prior work [42]. Cohort data, individual monitoring data (time series and risk rating), and source content (SM posts/behavior) that generated ratings were viewed via the dashboard.

### Measures

Mutltimedia Appendix 1 provides information regarding the measures by the reporter as well as the sample items.

#### Demographics

Participants self-reported their age, ethnicity/race, sexual orientation, and gender. In addition, school personnel reported their role in the school context and the years of experience in that role.

#### Implementation Outcomes

##### Acceptability

The 4-item acceptability of intervention measure [45] was used to assess school personnel’s perception of acceptability, including liking, approving, and welcoming use of the dashboard. Items were rated on a 5-point Likert scale (1=completely disagree and 5=completely agree). Prior psychometric evaluation suggested acceptable measurement model fit and high reliability [45], and internal consistency in this study was strong (α=.93).

##### Appropriateness

The 4-item intervention appropriateness scale [45] was used to assess school personnel’s perception of fit with items related to fit for the setting, applicability to their work, and a good match for the needs of the users. Items were rated on a 5-point Likert scale (1=completely disagree and 5=completely agree). Prior psychometric evaluation suggested acceptable measurement model fit and high reliability [45], and internal consistency in this study was excellent (α=.97).

##### Feasibility

The 4-item feasibility of intervention measure [45] was used to assess school personnel’s perception of acceptability, including possible, doable, and easy use of the dashboard. Items were rated on a 5-point Likert scale (1=completely disagree and 5=completely agree). Internal consistency in this study was strong (α=.91).

#### Implementation Determinants

##### Usability

School personnel completed the SUS following user testing. The SUS is a 10-item measure, with scores ranging from 0 to 100, with scores greater than 70 considered acceptable. The SUS is the best-researched and most sensitive usability measure available [35]. Internal consistency in this study was strong (α=.83).

##### Additional Determinants

Following user testing sessions, school personnel participants were asked a series of open-ended questions about what they saw as the positive aspects, the negative aspects, and specific suggestions for improvement based on other technologies with which they interacted. Questions focused on acceptability, feasibility, and appropriateness were asked to understand the reasons for their interview responses. Students completed the Preferences, Relationships, and Interventions using Social Media, a 22-item questionnaire developed by this team that assessed the use and frequency of SM platforms, priorities regarding intervention options, and open-ended questions around the ways to improve system alignment with the needs and expectations of students in their school.

### Data Analysis Plan

Descriptive statistics, including means and SDs, were calculated for quantitative measures. Qualitative content was coded using the Consolidated Framework for Implementation Research (CFIR) [46]. The CFIR is a commonly used framework that organizes constructs that have been associated with effective implementation. It has been widely used as a practical guide to evaluate implementation efforts in preparation for or during active studies [46]. The codebook template was used to understand the multilevel determinants of implementation. Determinants include aspects of the innovation (eg, evidence strength and relative advantage), outer context (eg, external policies and incentives), the inner organizational context (eg, implementation climate and tension for change ), characteristics of the individuals operating within target settings (eg, attitudes and efficacy), and process of change in the organization (eg, engagement strategies and change agents) [47-51]. School personnel interviews were audio recorded, transcribed, and coded with directed content analysis. In total, 4 coders were trained to conduct directed content analysis based on the CFIR codebook by reviewing the codebook and example codes, reviewing the school personnel’ s responses to each question from the same two transcripts, identifying potential codes, and independently coding. Consensus among the four coders for the two transcripts was achieved through open dialog [52]. Following consensus on the two transcripts, two coding team members who had completed the consensus coding were split into two groups. The remaining transcripts were coded independently and then the two groups met to review codes in consensus meetings. A consensus coding process was used to reduce biases, groupthink, and errors [53]. This coding approach was used, as many qualitative researchers consider it to be more valid for analyzing human communication, as it explicitly uses coding ambiguities to prompt discussion and increases confidence in complex data compared with interrater reliability [54].

## Results

### Participants

The demographic characteristics of the participants are included in Tables 2 and 3.

**Table 2:** Summary of demographics and clinical characteristics for student participants (N=111).

Characteristics		Students	
**Sex at birth, n (%)**			
	Male		40 (36.0)
	Female		71 (64.0)
	Intersex		0 (0.0)
**Gender, n (%)**			
	Male		40 (36.0)
	Female		71 (64.0)
	Transgender male		0 (0.0)
	Transgender female		0 (0.0)
Age (years), mean (SD)		16.5 (1.13)	
**Sexual orientation, n (%)**			
	Asexual		1 (0.9)
	Bisexual or pansexual		16 (14.4)
	Gay or lesbian		4 (3.6)
	Heterosexual or straight		83 (74.8)
	Other^a^		2 (1.8)
	Prefer not to say		5 (4.5)
**Ethnicity (Hispanic or Latino), n (%)**			
	Not Hispanic or Latino		103 (92.8)
	Hispanic, of Spanish Origin or Latino		5 (4.5)
	Prefer not to answer		3 (2.7)
**Race, n (%)**			
	White		63 (56.8)
	Black or African American		10 (9.0)
	American Indian or Alaska Native		0 (0.0)
	Asian		15 (13.5)
	Native Hawaiian or Other Pacific Islander		2 (1.8)
	Other, not specified above		7 (6.3)
	Unknown or prefer not to answer		2 (1.8)
	Multiracial		12 (10.8)
**Number of suicide attempts, n (%)**			
	0		106 (95.5)
	1		2 (1.8)
	2		3 (2.7)

^a^Sexual orientation: other: heterosexual and bicurious (n=1) and questioning (n=1).

**Table 3:** Summary of demographics for school personnel participants (N=9).

Characteristics			School personnel, n (%)
**Gender**			
	Male	2 (22)	
	Female	7 (78)	
	Other	0 (0)	
**Age (years)**			
	25-34	4 (44)	
	35-44	4 (44)	
	55-64	1 (11)	
**Ethnicity (Hispanic or Latino)**			
	Not Hispanic or Latino	9 (100)	
	Hispanic, of Spanish Origin or Latino	0 (0)	
**Race**			
	White	7 (78)	
	Black or African American	0 (0)	
	American Indian or Alaska Native	0 (0)	
	Asian	2 (22)	
	Native Hawaiian or Other Pacific Islander	0 (0)	
	Other, not specified above	0 (0)	
	Multiracial	0 (0)	
**Degree**			
	Bachelor’s	2 (22)	
	Master’s	7 (78)	
**Professional Role**			
	School counselor	3 (33)	
	Mental health counselor	2 (22)	
	School administrator	1 (11)	
	Other^a^	3 (33)	
**Years in role**			
	1-3	3 (33)	
	4-6	3 (33)	
	7-9	2 (22)	
	≥20	1 (11)	

^a^Professional role: other: school nurse (n=2) and community-based behavioral health partner (n=1).

### Implementation Outcomes

School personnel gave the student-monitoring dashboard moderate scores, on average, for acceptability (mean 3.69, SD 0.85; range 2.25-5.00), appropriate (mean 3.72, SD 1.09; range 2.00-5.00), and feasibility (mean 3.78, SD 0.75; range 2.25-4.75) of implementation in their setting, indicating school personnel viewed the student-monitoring dashboard as moderately appropriate for the school setting.

### Determinants of Implementation

To understand the reasons for ratings of core implementation outcomes, qualitative themes at the level of the innovation, outer setting, inner setting, individual characteristics, and engagement were summarized (Multimedia Appendix 2). The following three themes emerged: (1) compatibility with culture, values, and norms in the school setting; (2) additional attention needed to confidentiality and privacy; and (3) flexibility in the way to support students. The majority of the qualitative codes related to the first theme, that is, the organizational context, culture, resources, and structure (161/350, 46.0% of school personnel comments and 118/222, 53.2% of student comments). Specific comments highlight positive aspects of the system being compatible with culture and values/norms in the school setting. For example, a student indicated:

The system would be great if it helps a student personally and on their phone, and includes lots of student choice.

coded innovation characteristics, adaptability

However, both participant groups reported difficulty in managing confidentiality and privacy within this context and adequately managing the workflow. For example, 1 clinician stated:

A barrier to implementation in that we are not an organization that is accessible. This level of oversight is appealing in some ways and so I wonder if it creates an expectation of supervision or the impression of supervision where it’s not always available.

coded inner setting, available resources

A student’s perspective highlighted:

If people feel like they can’t be themselves on the social media because they don’t trust the system to keep their confidentiality then I don’t think they’d use it. If students didn’t use the social media then the system wouldn’t work at all.

coded outer setting, external policy

The second theme highlighted the need for careful attention to how information would be used within the school setting and remain confidential. Some expressed uncertainty about the extent to which machine learning can discern the complexities of unstructured text and nuanced communication occurring on SM platforms:

In today’s society the young generation us [sic] tend to make jokes about suicide in a way to relieve stress so I’m afraid something like that will be taken the wrong way.

coded innovation characteristics, evidence strength

School personnel and students noted wanting clarity on how the TES would impact internal communications and other external systems outside the school, including the district and outside resources (eg, therapists outside of the school and crisis responding).

The third theme relates to the potential for an approach similar to this to expand options for youth at risk. Overall, 34.0% of school personnel comments and 18.0% of student comments highlight the innovative aspects of using passive and ongoing data collection in this way. Themes of positive comments related to the relative perceived advantage of a technology-based solution compared with the status quo as well as the ability to provide individualized solutions and options for youth who appear distressed and/or suicidal. One student noted:

It would allow social media to be safer and less stressful for people who have a lot of anxiety about it.

coded innovation characteristics, relative advantage

Another student stated the asset of flexibility:

I think that the best thing this system could do was just be an option for people who are struggling to go and have someone or something that could help them and be there for them.

coded inner setting, available resources

### Prototype Interface Usability

#### Task Difficulty

Most tasks were estimated to be moderately easy (mean_range_ 3.78-4.22), with the exception of isolating a date range (mean_pre_ 3.00 and mean_post_ 2.22). Users found the task of free exploration and navigation of the dashboard, similar to or easier than they had anticipated. The majority of users found the task of identifying posts within a set time frame more difficult than they anticipated.

#### Task Success

All 100% of participants identified the students and the risk level. Two-thirds of the participants correctly identified previous suicide risk, and about half of the participants were able to flag concerning posts.

#### System Usability Scale Usability

School personnel’s scores on the platform ranged from 22.5 to 75, with a mean of 54.17 (SD 16.58), showcasing the divergent opinions from school personnel on the system’s overall usability and an overall unacceptable rating of current usability of the prototype dashboard interface (acceptable ratings >70). Feedback after each testing scenario and during the formal qualitative interview highlighted several common issues, themes, and needed modifications identified by the participants for a subsequent version of the interface (eg, difficulty in isolating specific periods within the interface).

## Discussion

### Principal Findings

In this study, high school students and school personnel provided feedback on implementation determinants and outcomes to facilitate the redesign of a TES to support suicide risk identification and prevention. Universal emotional health screening is recognized as an essential component of a multitier system of support and behavioral health framework [55]. Universal emotional health screening may facilitate the identification of undetected difficulties [56]; however, emotional health screening is rarely conducted in school settings because of feasibility, burden on school personnel, and lack of knowledge of best practices. A solution that supports accurate, ongoing, and passive screening for youth risk, clinical decision making, and improved communication and that fits within the school context would be a great asset toward facilitating identification and triage of students at risk for suicide. Through mixed qualitative and quantitative approaches, our study identified a number of strengths of the digital component of the Quinn Therapeutic TES. We additionally identified several challenges related to the school context and concerns regarding fit within workflow and the network of communications around protected health information such as suicidality. Three primary themes identified by students and school personnel were (1) compatibility with culture, values, and norms in the school setting; (2) additional attention needed to confidentiality and privacy; and (3) flexibility in the way to support students. With regard to compatibility with the culture, students, and school personnel highlighted that this approach aligned with the school’s value to support well-being and help achieve goals related to caring for the *whole student*. However, both stakeholders reported a need for additional information about the data and processes for analysis, interpretation, communication, and human responses were requested. Student concerns regarding confidentiality were centered on how school personnel would manage communications, not sharing data with a company for purposes of suicide prevention. Along with other researchers, before the widespread acceptance of a system that was supported by existing data sources such as SM, additional education regarding the validity of psychologically relevant data can be measured via SM language. Finally, both school personnel and students found appeal in TES flexibility, including the ability for multiple options for enrollment and strategies to support students.

Several researchers have suggested that digital innovations that rely on machine learning strategies similar to the one evaluated in this research would provide significant advances in the field [4,57-60]. In addition, programs that rely on suicide risk prediction algorithms have been deployed in the Veteran’s administration. This program, called Recovery Engagement and Coordination for Health—Veterans Enhanced Treatment, uses medical record data and applies machine learning to identify those at a statistically elevated risk for suicide or other adverse outcomes. At present, there is an active clinical trial of the program (NCT03280225). The evaluation of the application of machine learning algorithms to medical records for the prediction of suicide attempts has demonstrated good performance [61]. Few programs have been designed to provide rigorous evaluation of the usability and other implementation determinants for the use of a technology-based solution to universal suicide screening in the school context. Several limitations must be considered when interpreting the results. First, the sample of users was small, and not representative, as it was limited to participants from a small private school in an urban area. Second, we coded perceptions of the intervention implementation following scenario-based user testing, not actual implementation of the intervention, and, therefore, may not be valid for real-world implementation. Finally, we only included primary end users, that is, students and school personnel who would be involved in responding to suicide risk directly and, therefore, did not include other important stakeholders such as teachers and parents.

### Conclusions

Strategies to make suicide prevention efforts in high schools scalable, sustainable, and supportive may benefit from attention to how technology can facilitate and aid human efforts. This research evaluated a system that aggregates existing data sources—SM data—to provide ongoing monitoring of suicide risk based on machine learning algorithms. Primary users—high school students and school personnel—highlighted the potential advantages of providing individualized solutions and options for youth compared with the current suicide prevention strategy within the school (which included gatekeeper training, mental health awareness group, and onsite counseling support). However, the management of private and sensitive communications in the school context and limited functionality of the prototype dashboard dampened enthusiasm for widespread implementation. Although further investment in an improved user interface may improve some of the concerns, the large fundamental challenge facing this and similar TES is a lack of understanding and policy surrounding the privacy and use of sensitive communications in the school context. Widespread agreement on community norms and commonly accepted guidelines for how and when to use this sort of data will be necessary for the widespread adoption of any similar TES.

## Appendix

Multimedia Appendix 1 Measures, QT user function, and qualitative coding examples.

Multimedia Appendix 2 Qualitative coding reference table.

## Abbreviations

AUC: area under the curve
CFIR: Consolidated Framework for Implementation Research
HCD: human-centered design
SM: social media
SUS: system usability scale
TES: technology-enabled services
