# Supplementary material for: Implementation Determinants and Outcomes of a Technology-Enabled Service Targeting Suicide Risk in High Schools: Mixed Methods Study
Source: JMIR Ment Health. 2020 Jul 20;7(7):e16338. doi: 10.2196/16338 (PMC7399956; doi:10.2196/16338)
Supplement: Multimedia Appendix 1 [file mental_v7i7e16338_app1.docx]

Online Appendix: Student Measures.

| Purpose / Content: | Measure | Sample Question | Response Options/ Scale |
| --- | --- | --- | --- |
| To collect a variety of information to describe the participant characteristics, and to possibly use as control variables. Demographics, education, experience, professional background, role, etc. | Demographics | Which racial or ethnic group(s) best describe you? You may choose more than one. | • American Indian or Alaska Native  • Asian  • Black or African American  • Native Hawaiian or Other Pacific Islander  • White or Caucasian  • Other (Please specify) |
| To collect information on youth assessing 5 domains: emotional symptoms, conduct problems, hyperactivity/inattention, peer relationship problems, and prosocial behaviors | Strengths & Difficulties Questionnaire (SDQ) | I have one good friend or more | 0=Not True  1=Somewhat True  2=Certainly True |
| To collect information on different dimensions of suicidality, including lifetime suicide ideation and/or suicide attempt, frequency of ideation, threat of suicide attempt, and likelihood of suicidal behavior in the future. | Suicide-Behaviors Questionnaire-Revised (SBQR) | How often have you thought about killing yourself in the past year? | 0=Never  1=Rarely (1 time)  2=Sometimes (2 times)  3=Often (3-4 times)  4=Very Often (5+ times) |
| To collect information youth rating of problem behaviors. Areas of functioning assessed include interpersonal relations, broad psychopathological domains (e.g. internalizing/externalizing symptoms), functioning in job or school work, and use of leisure time. | Columbia Impairment Scale (CIS) | In general, how much of a problem do you think you have with: feeling unhappy or sad? | 0=No Problem  1  2= Some Problem  3=  4= Very Bad Problem  5= Not applicable/Don’t Know |
| To collect information on student perceptions of school climate along three dimensions: teaching and learning, relationships, and safety. | Positive Behavioral Interventions & Supports (PBIS) School Climate Survey: Middle/High | School is a place at which I feel safe. | 0 = Strongly Disagree  1 = Somewhat Disagree  2 = Somewhat Agree  3 = Strongly Agree |
| To collect information on overall social media use and attitudes, preferences, relationships and interventions using social media. | Preferences, Relationships & Interventions Using Social Media (PRISM) | What would you want to be sure the system included? | 0=Privacy controls to turn on and off needs  1=Assurances of confidentiality and privacy  2=Choices about how my indication of stress was handled by school staff  3=Other |
|  | Open-ended Question (included at the end of the PRISM) | What would be the best way this system could support you and the well-being of students in your school? |  |

School Personnel Measures.

| Purpose / Content: | Measure | Sample Question | Response Options/ Scale |
| --- | --- | --- | --- |
| To collect a variety of information to describe the participant characteristics, and to possibly use as control variables. Demographics, education, experience, professional background, role, etc. | Demographics | What is your most advanced degree? | 1= Associate’s Degree  2= Bachelor’s Degree  3= Master’s Degree  4= Doctoral Degree |
| The purpose of this scale is to measure usability of the ASSESS student-monitoring dashboard. The System Usability Scale (SUS) is a simple, ten-item scale giving a global view of subjective assessments of usability. | System Usability Scale (SUS) | I think that I would like to use this system frequently | 1= Strongly Disagree  2= Disagree  3= Neither Agree nor Disagree  4= Agree  5= Strongly Agree |
| The purpose of this scale is to measure acceptability, appropriateness, and feasibility of the student-monitoring dashboard. | Acceptability, Appropriateness & Feasibility Scale (AAFS) -- *Acceptability of Intervention Measure (AIM)* | This system is appealing to me | 1= Completely Disagree  2= Disagree  3= Neither Agree nor Disagree  4= Agree  5= Completely Agree |
| The purpose of this scale is to measure acceptability, appropriateness, and feasibility of the student-monitoring dashboard. | Acceptability, Appropriateness & Feasibility Scale (AAFS) -- *Intervention Appropriateness Measure (IAM)* | This system seems applicable | 1= Completely Disagree  2= Disagree  3= Neither Agree nor Disagree  4= Agree  5= Completely Agree |
| The purpose of this scale is to measure acceptability, appropriateness, and feasibility of the student-monitoring dashboard. | Acceptability, Appropriateness & Feasibility Scale (AAFS) -- *Feasibility of Intervention Measure (FIM)* | This system seems doable | 1= Completely Disagree  2= Disagree  3= Neither Agree nor Disagree  4= Agree  5= Completely Agree |
|  | Open-ended Questions (included at the end of the Interview) | 1. We’d like to hear your general impressions about this prototype of an online system that would monitor student safety *(need probes here)*    1. *Anything confusing, strange, unusual or difficult to understand?*    2. *Did you find it difficult or easy to interpret screens?*    3. *Overall feeling navigating through dashboard?* 2. Which components of the dashboard did you find most/least impactful, and why? 3. What aspects of the student monitoring dashboard do you think will be helpful in improving student safety monitoring? 4. Have you used or interacted with other monitoring systems (e.g. EHRs) that are similar to this? If so, how does this system compare?    1. *Ease of use?*    2. *Visual clarity?*    3. *Other functionality?* 5. When thinking about implementing a system like this,    1. How would you prefer to be alerted about changes in student status?  *(probes – what are the options we could be asking about?)*    2. What would an acceptable number be of alerts per day?    3. Who would be responsible for managing the alerts?    4. If you needed to refer a youth to a community agency, based on the information in this system, what would be the best way to communicate with them? 6. What do you see as the biggest barriers or challenges to the successful use of a program like this on a larger scale?    1. *Individual level barriers? (time, energy, training, etc.)*    2. *Institutional/systems level barriers? (resources, administrative buy-in, students/parents)* 7. Please describe why you think the student monitoring dashboard will or will not be helpful to schools that are interested in assessing the suicide risk status of their students.    1. *Can you tell me a bit more…?*    2. *Please explain …* 8. What aspects of your school context *(identify what we mean about this so that we can describe to the interviewee)* would make programs like this more or less relevant and effective? 9. Do you believe that a student safety monitoring program would be beneficial?    1. *Why or why not?*    2. *Where are the opportunities to improve the existing system?* 10. Describe your impressions about how easy it is to implement this system into your school? What factors impact the ease with which this system could be implemented? 11. What are the biggest needs school professionals have when it comes to assessing and responding to adolescent suicide risk? 12. Do you have any suggestions for improving the dashboard or implementation approach? 13. Do you have any additional comments or questions regarding the tasks or the overall prototype for the student-monitoring dashboard? |  |

Image Glossary of User Functions.

| Timeline Chart | 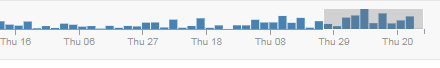 |
| --- | --- |
| Search Bar | 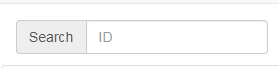 |
| Identified Suicide Risk Tabs | 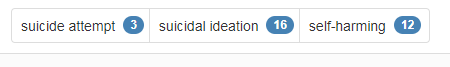 |
| Sentiment Graph | 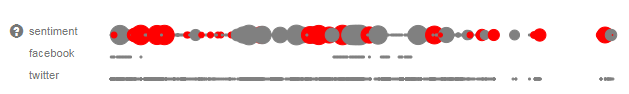 |
| Self-Stated Diagnosis & Suicide Attempt Tags | 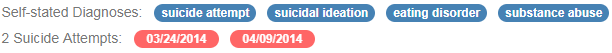 |
| Risk-Level Graph | 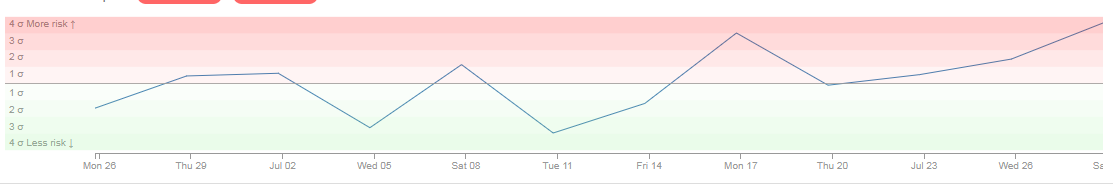 |
| Social Media Post Content | 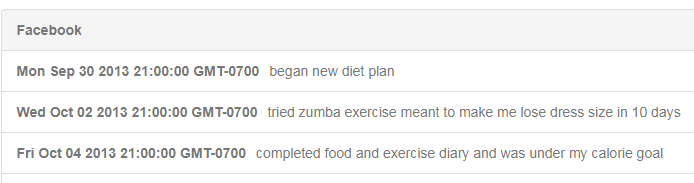 |

Primary codes and subcodes for Implementation Determinants

| Qualitative Coding Data | | | |
| --- | --- | --- | --- |
| Code | Subcode^a^ | School Personnel (n=350) | Student (n=222) |
| Innovation Characteristics | Evidence Strength and Quality  Relative Advantage  Adaptability  Complexity  Design Quality and Packaging | *"Our main counselling office downstairs is really centrally located and we know its visibility is sometimes an obstacle to students coming in and seeking help and so this could potentially be a way for them to signal that they need help or to solicit some follow up without having to physically walk into the door. And I think we know that there are students out there that are at risk but we are not aware of so it’s appealing from that perspective. "* | *"It’s definitely important for the user to be aware of the different choices, so I think it’s good for the system to provide options based on our behavior, and for us to choose what’s best for us."* |
| Outer Setting | Needs and Resources  External Policy and Incentives | *"The reason why a district won’t do this is politics. The reason that they won’t adopt this is because they’re afraid that they’re gonna get information and miss it and then they will be held liable. That’s really the only reason that you wouldn’t do this"* | *"I think assurance of confidentiality and privacy would be a good bet because if people feel like they can't be themselves on the social media because they don't trust the system to keep their confidentiality then I don't think they'd use it. If students didn't use the social media then the system wouldn't work at all."* |
| Inner Setting | Networks and Communications  Culture  Tension for Change  Compatibility  Relative Priority  Learning Climate  Leadership Engagement  Available Resources  Access to Knowledge/Info | *"So, and again, what level of responsiveness does that open up for me as a counselor if it rolls into my email at 11.30 at night, I may have not seen it. I don’t know if that person is a 911 call level of risk of follow up with them before school or see them sometime in the next week kind of risk. So I wonder about the level of information it’s giving me but not a lot of background info or context. And there’s definitely students I know are risks already that I think about when I wake up in the middle of the night and being able to know that there’s a set of eyes, in a way, helpful"* | *" It is important that people know they have convenient resources that can help their depression, anxiety, etc. but these services should by no means be forced upon a person"* |
| Individual Characteristics | Knowledge/ Beliefs Innovation  Self-Efficacy  Individual Identification with Org.  Other Personal Attributes | *“It’s such a complex matter, it's like trying to figure out...should adults have access to that, is that private, you know, as a therapist, I, would not want to have access to my client's Facebook. It's not appropriate. So, it's super complicated, so would it be helpful, maybe, maybe there's a tool that would be helpful in understanding suicide risk, but, this particular tool would not, in its format currently, would not be something that I would suggest that I or my colleagues use, necessarily"* | *"Students don’t like to be told about their problems, nor which solutions work the best since we already have to deal with adults telling us in what way we should act, but instead create atmospheres such as advisory, where students feel safe to tell an advisor about something in their life that is troubling them".* |
| Engaging | Key Stakeholders  Students | *"I think some students would really take a part in that, this is extra help, you’re really wanting to help me instead of ‘you’re just checking in with me because you have to’. I think students want to talk about it, but they just don't feel like they can, or who can they turn to, that’s why social media is a big thing."* | *"If people feel like they can't be themselves on the social media because they don't trust the system to keep their confidentiality then I don't think they'd use it. If students didn't use the social media then the system wouldn't work at all."* |
| ^a^Subcodes used reflect a select ion of constructs taken from Consolidated Framework for Implementation Research (CFIR) | | | |
